# A daily high-resolution (1 km) human thermal index collection over the North China Plain from 2003 to 2020

**DOI:** 10.1038/s41597-023-02535-y

**Published:** 2023-09-18

**Authors:** Xiang Li, Ming Luo, Yongquan Zhao, Hui Zhang, Erjia Ge, Ziwei Huang, Sijia Wu, Peng Wang, Xiaoyu Wang, Yu Tang

**Affiliations:** 1 School of Geography and Planning, and Guangdong Key Laboratory for Urbanization and Geo-simulation, Sun Yat-sen University, Guangzhou, 510006 China; 2 Institute of Environment, Energy and Sustainability, The Chinese University of Hong Kong, Hong Kong, China; 3 Key Laboratory of Watershed Geographic Sciences, Nanjing Institute of Geography and Limnology, Chinese Academy of Sciences, Nanjing, 210008 China; 4 Dalla Lana School of Public Health, University of Toronto, Toronto, Ontario M5T 3M7 Canada

**Keywords:** Atmospheric science, Climate change

## Abstract

Human-perceived temperature (HPT) describes the joint effects of multiple climatic factors such as temperature and humidity. Extreme HPT events may reduce labor capacity and cause thermal discomfort and even mortality. These events are becoming more frequent and more intense under global warming, posing severe threats to human and natural systems worldwide, particularly in populated areas with intensive human activities, e.g., the North China Plain (NCP). Therefore, a fine-scale HPT dataset in both spatial and temporal dimensions is urgently needed. Here we construct a daily high-resolution (~1 km) human thermal index collection over NCP from 2003 to 2020 (HiTIC-NCP). This dataset contains 12 HPT indices and has high accuracy with averaged determination coefficient, mean absolute error, and root mean squared error of 0.987, 0.970 °C, and 1.292 °C, respectively. Moreover, it exhibits high spatiotemporal consistency with ground-level observations. The dataset provides a reference for human thermal environment and could facilitate studies such as natural hazards, regional climate change, and urban planning.

## Background & Summary

Under global warming, the frequency and intensity of extreme temperature events (e.g., heatwaves and cold spells) are increasing in most parts of the world^1–5^. They pose severe impacts on human society and the ecological systems^6–10^, putting more people at risk of extremely hot or cold^11,12^. For example, heatwave led to more than 70,000 deaths in Europe in 2003 and 55,000 deaths in Russia in 2010^13,14^. Extreme cold events also pose a great threat to human health and often cause a higher proportion of deaths than extreme heat^15,16^.

Human-perceived temperature (HPT) has attracted much attention in recent years due to its close connection with human health^17,18^. HPT is not only affected by air temperature but also by other meteorological variables (e.g., humidity and wind speed). For instance, HPT would become higher on hot days with high humidity while becoming lower on cold days under the additional influence of wind^19–22^. Relevant studies revealed that discomfort HPT could impact human health, such as respiratory distress and reduced skin evaporation, and significantly increase the health risks and mortality in heat- and cold-exposed populations under the joint effects of multiple meteorological factors^23–25^. Li *et al*.^26^ showed that global HPT increases faster than the actual air temperature. Faster increases in HPT were also confirmed by regional studies^27,28^. However, previous studies mainly quantitated the human thermal environment and human heat exposure assessment based on meteorological station data and homogeneous climatic datasets^29–32^. These station-based or raster-based datasets have a coarse spatial resolution (e.g., 0.5° × 0.5° or 2.5° × 2.5°) and insufficient temporal resolution (e.g., monthly), which cannot meet the requirements of long time series, full regional coverage, and fine spatial scale studies^28^. A seamless dataset of HPT with high temporal frequency and spatial resolution is still lacking and sorely needed.

A better understanding of the thermal environment helps prevent or mitigate heat/cold stress effectively^18^. Various HPT indices have been proposed to investigate human thermal stress previously. For example, the wet-bulb temperature (WBT), effective temperature (ET), and heat index (HI) have been widely used for quantitative assessment of human thermal environment research^17,28,33–35^. However, there is still a lack of unified evaluation standards or comprehensive datasets for assessing the human thermal environment and stress^36^. Therefore, it is imperative to develop a new collection with multiple indices for evaluating the thermal environment at a fine scale (e.g., at daily frequencies, and ~0.00833° spatial resolution in a geographical coordinate system, which is equal to around 1 km near the equator).

Previous studies have projected that the morbidity and mortality induced by extreme temperatures may continue to rise, especially in populated Asia^37–39^. In particular, the North China Plain (NCP), as one of the most populated and urbanized areas of the world, is suffering from severe thermal stress^40,41^. It is located in a typical East Asian monsoon region (113°E–121°E and 34°N–41°N)^40,42^, and spans eleven provincial units of China, including one of the regions with the most profound human activities in China, i.e., the Beijing-Tianjin-Hebei region^43^. NCP has a total population of 0.3 billion, which accounts for around one-fifth of the population of the country^44^. The population exposed to heatwaves in this area is up to 0.66 million persons/day during 1961–2015^45^. By examining the long-term changes of major urbanization agglomerations in China, Wang *et al*.^46^ indicated that HPT in northern China grew faster than that in the south, particularly in NCP. Zhang *et al*.^47^ have generated a 1-km-resolution dataset of monthly human thermal index collection (HiTIC-Monthly) over China during 2003–2020. However, this monthly dataset is not capable of detecting extreme weather events such as heatwaves and cold spells, which are usually defined based on daily temperatures. The lack of a daily HPT dataset limits accurate heat stress-related studies in this region, and a multi-index dataset of human thermal stress at a daily scale over NCP is highly essential.

**To fill the aforementioned research gap, this study aims to construct a high spatial resolution (1 km × 1 km) human thermal index collection at a daily scale over NCP from 2003 to 2020 (i.e., HiTIC-NCP)**. The 12 widely-used indices include Surface Air Temperature (SAT), indoor Apparent Temperature (AT_in_), outdoor shaded Apparent Temperature (AT_out_), Discomfort Index (DI), Effective Temperature (ET), Heat Index (HI), Humidex (HMI), Modified Discomfort Index (MDI), Net Effective Temperature (NET), Wet-Bulb Temperature (WBT), simplified Wet-Bulb Globe Temperature (sWBGT), and Wind Chill Temperature (WCT). Compared with our previous HiTIC-Monthly dataset, the current HiTIC-NCP dataset has improved the temporal resolution from a monthly to a daily scale. It also incorporates different predictors by including new variables such as daily precipitable water vapor, which reflect the high-frequency (day-to-day) fluctuations of atmospheric humidity that induces excessive heat stress as perceived by human body. This study is thus of great significance for regional climate change, urban planning, urban thermal environment, and public health research.

## Methods

### Overview

**Fig. 1:**
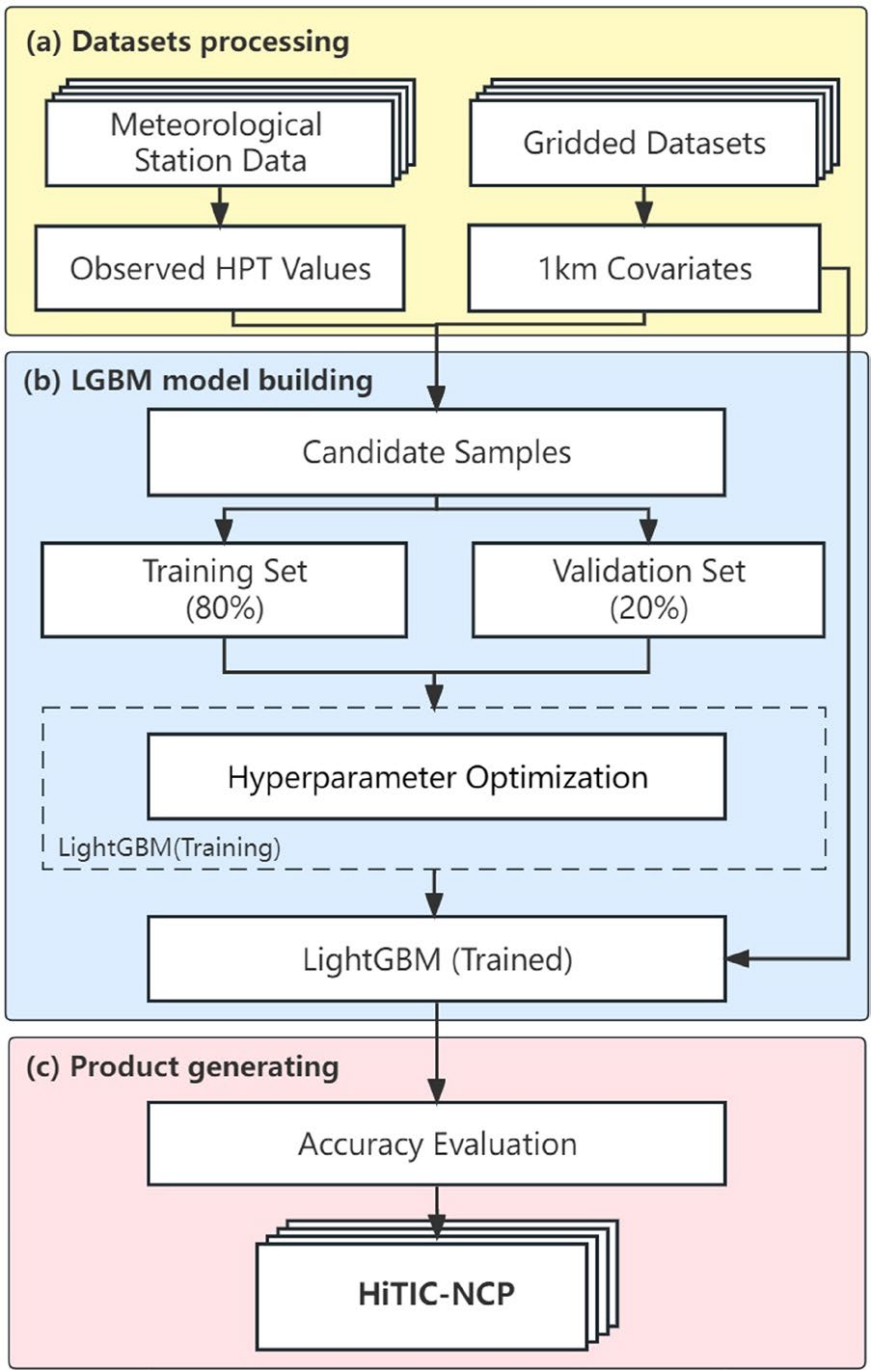
Framework of generating the daily human thermal index collection over the North China Plain (HiTIC-NCP). HPT: Human-perceived Temperature; LightGBM: Light Gradient Boosting Machine.

The data used in this study mainly include meteorological station observations and four gridded datasets. The Light Gradient Boosting Machine (LightGBM) algorithm proposed by Ke *et al*.^48^ is used to produce 12 human thermal indices. The workflow of this study is shown in Fig. 1. First, we derived daily HPT indices from daily values of temperature, humidity, and wind speed based on station observations, and preprocessed the daily gridded datasets. We extracted the covariate values at meteorological stations from gridded datasets to form candidate samples (which include station-level HPTs and their co-located covariate values), then randomly selected 80% of the samples as a training set and 20% of that as a validation set. Hyperparameter optimization based on a 5-fold cross-validation was used to determine the optimal parameters of the model by the training set, and a systematic evaluation of model performance was conducted based on the validation set. Finally, a collection of 12 HPTs was generated by the trained LightGBM model. The detailed data and methods are described as follows.

### Meteorological observations

**Fig. 2:**
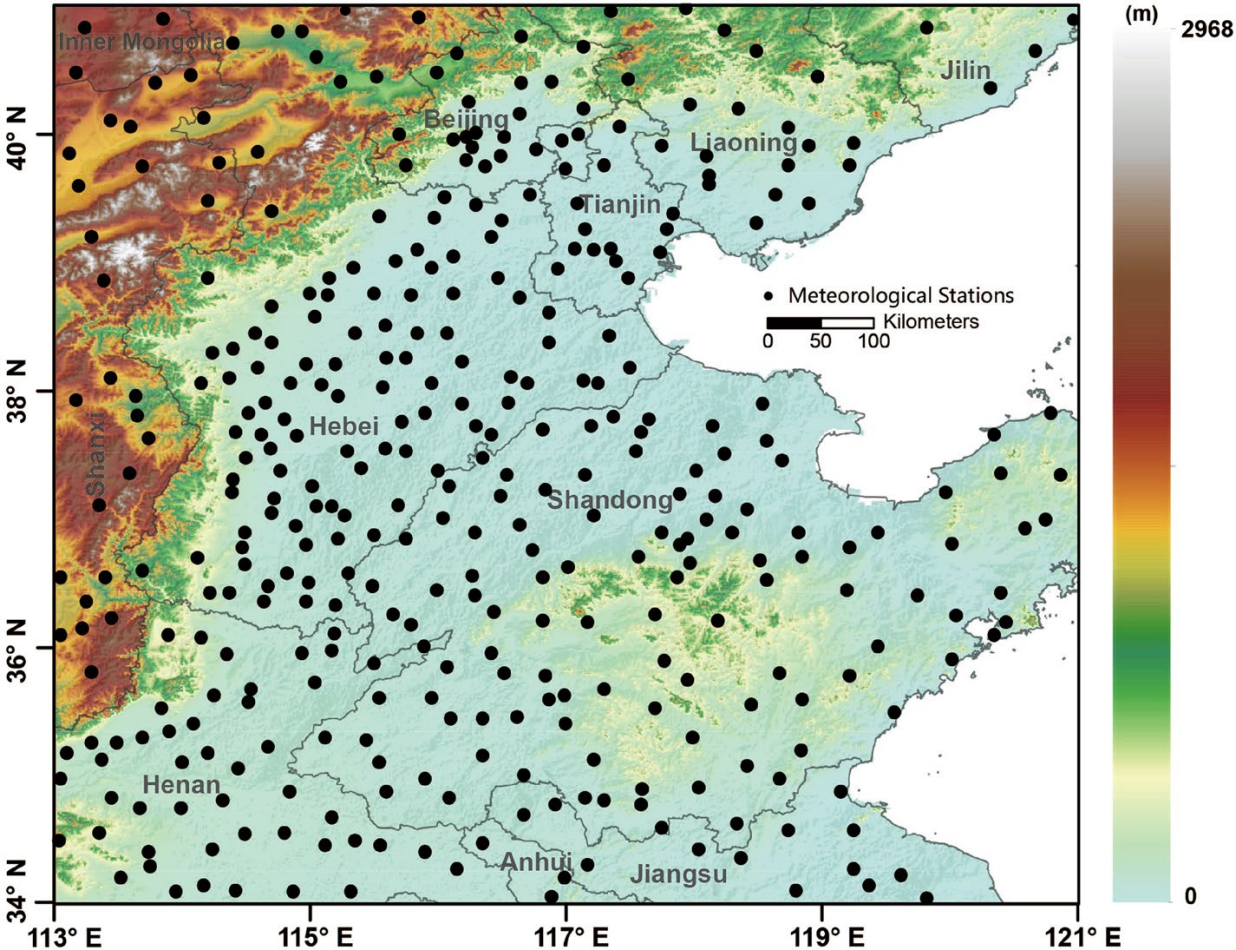
Study area and the spatial distribution of meteorological stations in the North China Plain (NCP). The shaded color indicates elevation, and the black dots denote the stations.

The station-observed daily data including surface air temperature, relative humidity, and wind speed from 2003 to 2020 were collected from the China Meteorological Data Service Center (CMDSC, http://data.cma.cn). All station data have been strictly quality-controlled based on the method provided by Xu *et al*.^49^, including homogenizing climate data and processing outliers in the data series. The observational network includes 374 stations across NCP (Fig. 2). In this study, we calculated daily human-perceived temperature from meteorological observations and extracted the covariate values from gridded datasets at the same locations. The data were stored in the form of one per station per day, then processed and divided into training (80%) and validation (20%) sets for model calibration. The calculation of the 12 human thermal indices is described in Table 1.

**Table 1:** Equations of the 12 thermal indices.

Name	Human Thermal index	Equation	Reference
AT_in_	Apparent Temperature (indoors)	$A{T}_{in}=-1.3+0.92\times T+2.2\times {E}_{a}$	Steadman^71^
AT_out_	Apparent Temperature (outdoors, in the shade)	$A{T}_{out}=-2.7+1.04\times T+2\times {E}_{a}-0.65\times V$	Steadman^72^
DI	Discomfort Index	$DI=0.5\times WBT+0.5\times T$	Epstein and Moran^73^
ET	Effective Temperature	$ET=T-0.4\times \left(T-10\right)\times \left(1-0.001\times RH\right)$	Gagge *et al*.^74^
HI	Heat Index	$$\begin{array}{c}{HI}^{\ast }=-8.784695+1.61139411\times T-2.338549\times RH-0.14611605\times T\times RH-1.2308094\,\times \\ 1{0}^{-2}\times {T}^{2}-1.6424828\times 1{0}^{-2}\times R{H}^{2}+2.211732\times 1{0}^{-3}\times {T}^{2}\times RH+7.2546\times 1{0}^{-4}\times T\times R{H}^{2}\,+\\ 3.582\times 1{0}^{-6}\times {T}^{2}\times R{H}^{2}\end{array}$$	Rothfusz and Headquarters^75^
HMI	Humidex	$HMI=T+0.5555\times \left(0.1\times {E}_{a}-10\right)$	Masterton and Richardson^76^
MDI	Modified Discomfort Index	$MDI=0.75\times WBT+0.38\times T$	Moran *et al*.^77^
SAT	Surface Air Temperature	Air temperature at 2-meter height	/
sWBGT	Simplified Wet-bulb Temperature	$sWBGT=0.567\times T+0.0393\times {E}_{a}+3.94$	Willett and Sherwood^78^
WBT	Wet-bulb Temperature	$WBT=T\times {\rm{atan}}\left(0.151977\times {\left(RH+8.313659\right)}^{0.5}\right)+{\rm{atan}}\left(T+RH\right)-{\rm{atan}}\left(RH-1.676331\right)+0.00391838\times R{H}^{1.5}\times {\rm{atan}}\left(0.02301\times RH\right)-4.686035$	Stull^79^
NET	Net Effective Temperature	$NET=37-\frac{37-T}{0.68-0.0014\times RH+\frac{1}{1.76+1.4\times {V}^{0.75}}}-0.29\times T\times \left(1-0.01\times RH\right)$	Houghton *et al*.^80^
WCT	Wind Chill Temperature	$WCT=13.12+0.6215\times T-11.37\times {\left(V\times 3.6\right)}^{0.16}+0.3965\times T\times {\left(V\times 3.6\right)}^{0.16}$	Osczevski and Bluestein^81^

*T* is air temperature (°C) at 2-meter height, *RH* is relative humidity (%), *V* is wind speed (m/s), and *E*_*a*_ is actual vapor pressure (kPa). The asterisk (*) means that an adjustment is needed. The unit of all human thermal indices is degree Celsius (°C).

### Gridded datasets

Four gridded datasets with a total of seven independent variables (Table 2) are used as input in the LightGBM model. These variables include land surface temperature (LST), population density, elevation, slope, aspect, precipitable water vapor, and day of the year.

**Table 2:** Gridded datasets and covariates used in this study.

Category	Spatial Resolution	Temporal Resolution	Source	Reference
Land surface temperature	1 km	Daily	10.25380/iastate.c.5078492	Zhang *et al*.^51^
Population density	Aggregated to 1 km	Annual	https://developers.google.com/earth-engine/datasets/catalog/WorldPop_GP_100m_pop	Gaughan *et al*.^56^
Elevation, slope, aspect	Aggregated to 1 km	/	https://developers.google.com/earth-engine/datasets/catalog/MERIT_DEM_v1_0_3	Yamazaki *et al*.^57^
Precipitable water vapor	1 km	Daily	https://developers.google.com/earth-engine/datasets/catalog/MODIS_061_MCD19A2_GRANULES#dois	Lyapustin and Wang^82^
Day of the year	/	/	/	/

**Table 3:** Overall R^2^, MAE, and RMSE values of the 12 human thermal indices over NCP from 2003 to 2020.

Index	R²	MAE (°C)	RMSE (°C)
AT_in_	0.989	0.946	1.246
AT_out_	0.988	1.052	1.390
DI	0.989	0.833	1.104
ET	0.987	0.598	0.785
HI	0.987	1.086	1.451
HMI	0.990	1.174	1.558
MDI	0.989	0.961	1.274
SAT	0.987	0.941	1.238
sWBGT	0.990	0.698	0.926
WBT	0.989	0.814	1.082
NET	0.975	1.373	1.837
WCT	0.984	1.167	1.614

LST is closely related to near-surface air temperature^50^. In this study, we adopted an LST dataset named MODIS-like LST^51^. This dataset was developed based on the Moderate Resolution Imaging Spectroradiometer (MODIS) LST dataset and the gap-filling algorithm^51^ to make it seamless at a global scale in 1 km grids from 2003 to 2020. The average root mean squared error (RMSE) of this LST dataset is 1.80–2.03 °C and 1.23–1.45 °C for daytime and nighttime temperatures, respectively. The daily mean LST was calculated by averaging the corresponding daytime and nighttime LSTs. In addition, population distribution and regional population density are demonstrated to affect regional temperature^52–55^, and we used a high-accuracy population density dataset provided by the WorldPop project team as a predictor^56^.

The Multi-Error-Removed Improved-Terrain (MERIT) Digital Elevation Model (DEM)^57^ with a spatial resolution of 90 m was used in this study to extract slope and aspect. Compared with previous DEM data (e.g., SRTM and AW3D-30m), the errors of MERIT DEM are significantly reduced, and the accuracy in the flat area has been effectively improved^57–59^. In addition, precipitation has been proven to affect air temperature and humidity^60,61^. Therefore, a global daily precipitable water vapor dataset with a spatial resolution of 1 km was used.

### The LightGBM machine learning algorithm

LightGBM is a gradient boosting decision tree (GBDT) based machine learning algorithm. Compared with other algorithms based on GBDT such as XGBoost and pGBRT, LightGBM has improved and optimized by the Gradient-based One-Side Sampling (GOSS) and Exclusive Feature Bundling (EFB) technologies, which allow LightGBM to better retain samples with large gradients without significantly altering the original data shape and focus more on undertrained samples^48^. LightGBM consumes much less memory and improves training efficiency by more than 20 times while maintaining nearly the same accuracy^48^. Due to its superior degree of generalizability, extremely fast processing speed, and high accuracy, LightGBM has been widely applied in different fields (e.g., climatology, air pollution, social psychology)^47,62–67^. As shown in Fig. 1, the daily gridded datasets were fed to the trained LightGBM model for HPT prediction.

### Hyperparameters tunning

The accuracy of machine learning relies heavily on parameterization^68^. Hyperparameter optimization configures the model by searching for various model hyperparameters and selecting a subset that achieves the best performance on the given dataset. The grid search-based hyperparameters tunning method was adopted in our study^69^.

### Accuracy evaluation

Three statistical metrics were used to assess the performance of the training model, including the determination coefficient (R^2^), root mean absolute error (MAE), and RMSE. The R^2^, RMSE, and MAE values are computed as follows:

1 $$\begin{array}{c}{R}^{2}=1-\frac{{\sum }_{i=1}^{N}{\left({x}_{oi}-{x}_{pi}\right)}^{2}}{{\sum }_{i=1}^{N}{\left({x}_{pi}-\overline{{x}_{o}}\right)}^{2}}\end{array}$$

2 $$\begin{array}{c}RMSE=\sqrt{\frac{1}{N}{\sum }_{i=1}^{N}{\left({x}_{oi}-{x}_{pi}\right)}^{2}}\end{array}$$

3 $$\begin{array}{c}MAE=\frac{1}{N}{\sum }_{i=1}^{N}\left|{x}_{oi}-{x}_{pi}\right|\end{array}$$

where *N* represents the number of data points, *x*_*p*_ and *x*_*o*_ are the *i*_*th*_ prediction and observation values, respectively.

## Data Records

**Fig. 3:**
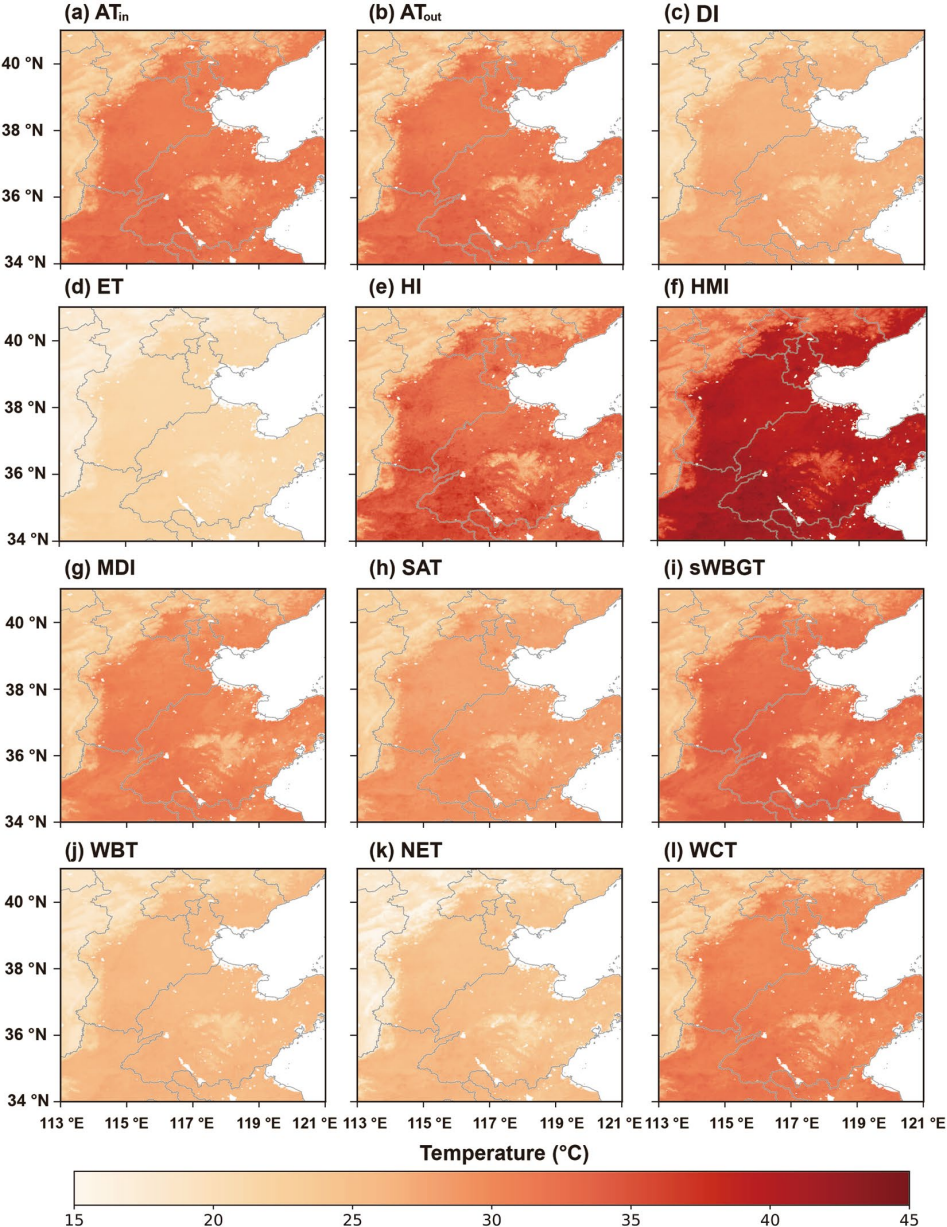
Spatial distribution of the 12 human thermal indices over NCP on August 13, 2013: (**a**) AT_in_, (**b**) AT_out_, (**c**) DI, (**d**) ET, (**e**) HI, (**f**) HMI, (**g**) MDI, (**h**) SAT, (**i**) sWBGT, (**j**) WBT, (**k**) NET, and (**l**) WCT.

**Fig. 4:**
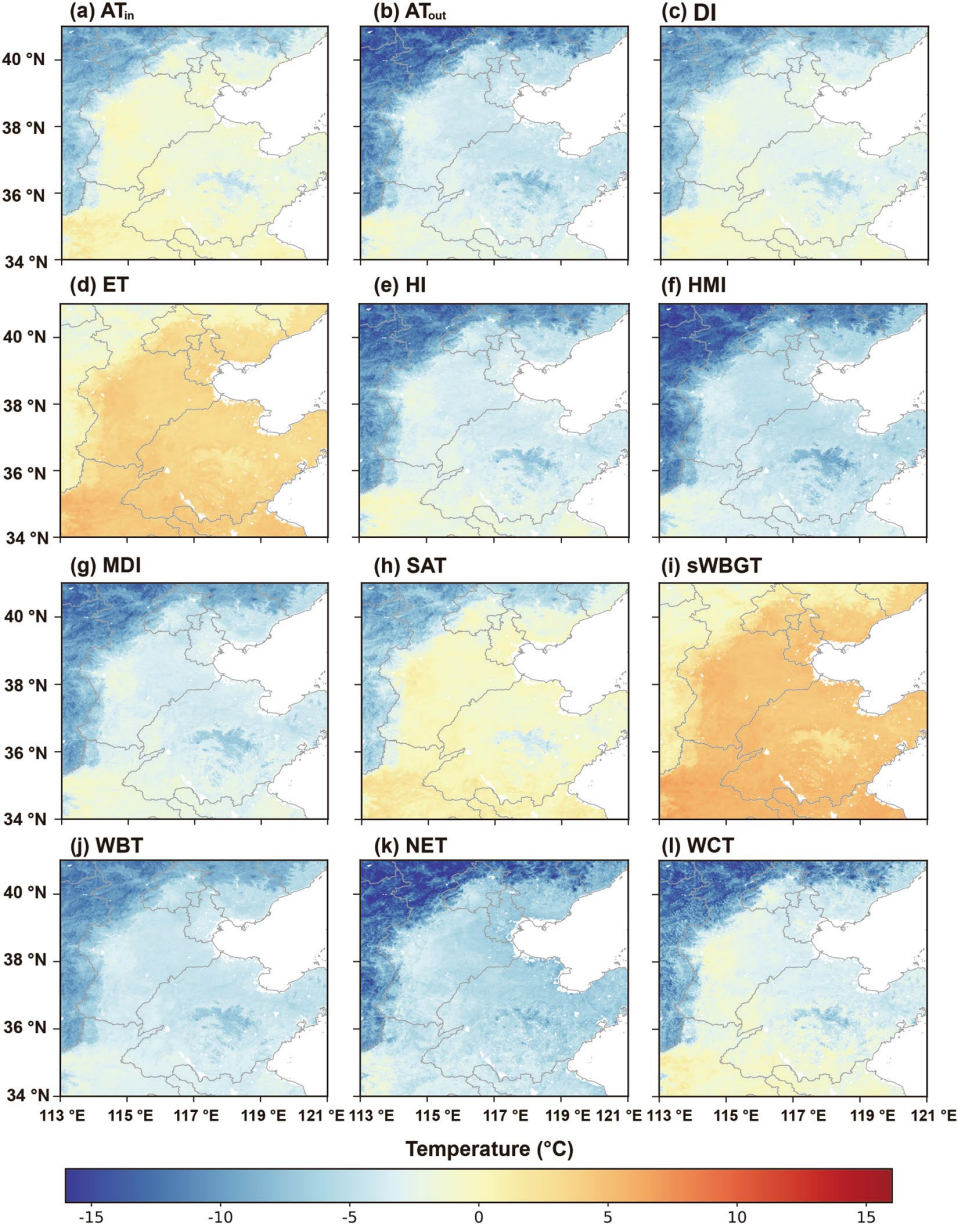
As Fig. 3 but on January 3, 2008.

The high spatial resolution (1 km) human thermal index collection at a daily scale from 2003 to 2020 over NCP (HiTIC-NCP) shows a high consistency between observations and predictions, and the product has respectable accuracies. HiTIC-NCP contains 216.rar files, with individual files decompressed into the NetCDF format. The dataset is available on the general-purpose repository Zenodo, at 10.5281/zenodo.7528001^70^. This dataset is stacked by year and each stack consists of daily images by day of the year in the NetCDF format. The unit of the dataset is 0.01 degree Celsius (°C), and the values are stored in an integer data type (Int16) to save storage space, and thus need to be divided by 100 to get the values in degree Celsius when in use. The geographic coordinate system of the dataset is World Geodetic System 1984 (WGS84) Coordinate System. The spatial distribution of the 12 human thermal indices on August 13, 2013, one of the severest heat stress days in the history of NCP, and January 3, 2008, when the sharpest snowstorm struck NCP, are shown as examples in Figs. 3, 4.

## Technical Validation

### Overall accuracy of 12 predicted human thermal indices

**Fig. 5:**
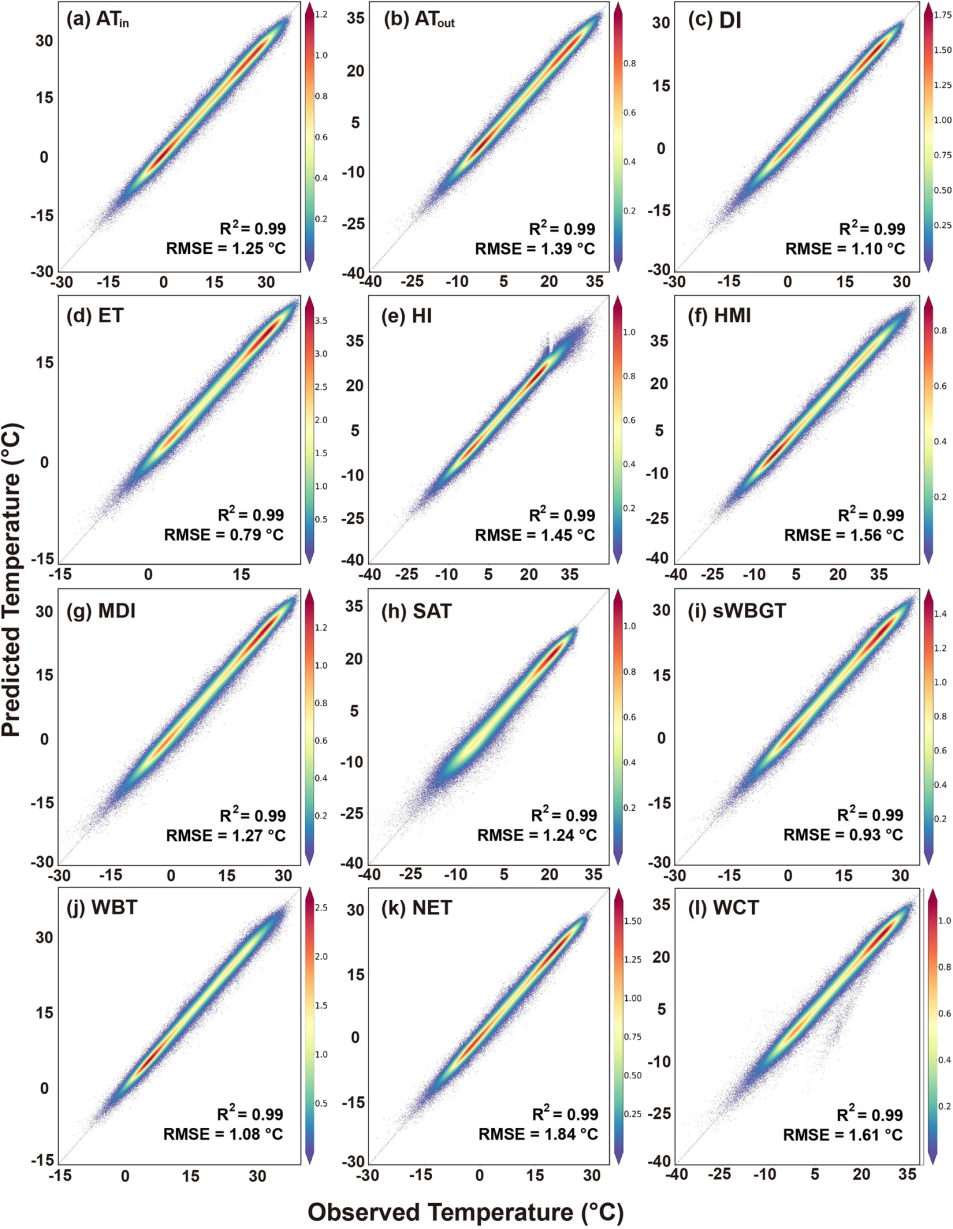
Scatter plots of predicted versus observed daily human thermal indices over NCP from 2003 to 2020: (**a**) AT_in_, (**b**) AT_out_, (**c**) DI, (**d**) ET, (**e**) HI, (**f**) HMI, (**g**) MDI, (**h**) SAT, (**i**) sWBGT, (**j**) WBT, (**k**) NET, and (**l**) WCT. Colors mean the density of data points, and the black dashed line is the 1:1 line.

We assessed the overall accuracy of 12 human thermal indices over NCP during 2003–2020 in terms of R^2^, MAE, and RMSE. All indices exhibit desirable performance, with the averaged R^2^, MAE, and RMSE being 0.987, 1.292 °C, and 0.970 °C, respectively. It demonstrates that the LightGBM model has good effectiveness in the prediction of human thermal indices at a daily scale. Figure 5 displays the scatter plots of observations versus predictions of 12 human thermal indices. The closer the scattered points are to the 1:1 line, the better agreement between the predicted and observed values.

**Fig. 6:**
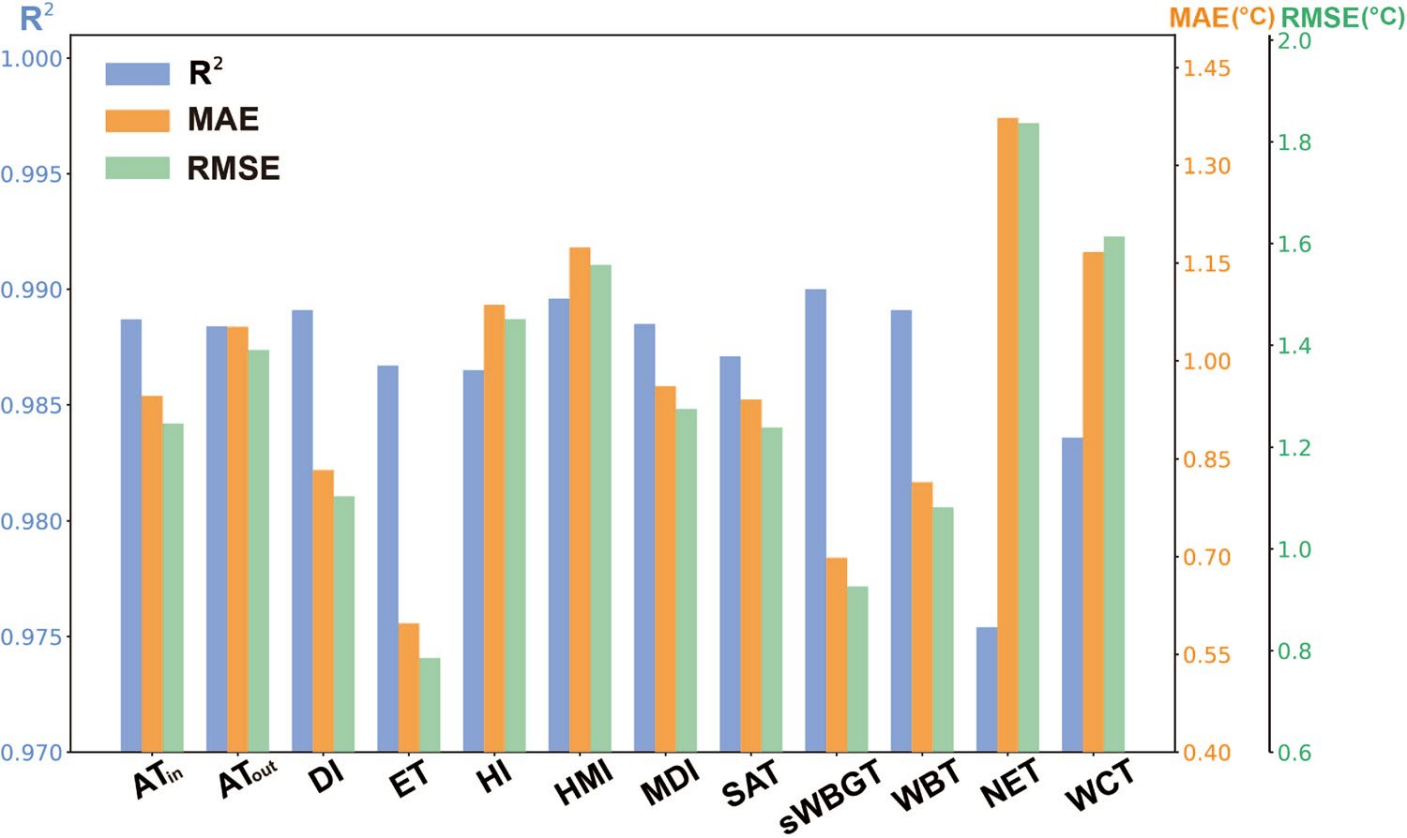
R^2^, MAE, and RMSE values of 12 predicted human thermal indices over NCP from 2003 to 2020.

The R^2^, MAE, and RMSE values of 12 predicted thermal indices during 2003–2020 are shown in Fig. 6. The R^2^ values range from 0.975 to 0.990, the MAE values are all smaller than 1.4 °C, and the RMSE values are smaller than 1.9 °C (Table 3). Particularly, sWBGT has the highest R^2^ values of 0.990, followed by HMI, WBT, and DI, and its MAE and RMSE values are 0.698 °C and 0.926 °C, respectively. ET is with an R^2^ of 0.987 and has the lowest MAE and RMSE values (i.e., 0.598 °C and 0.785 °C, respectively). WCT and NET exhibit the highest MAEs (i.e., 1.17 and 1.37 °C, respectively) and RMSEs (i.e., 1.61 and 1.84 °C, respectively). It is likely caused by the involvement of wind speed in the calculation of WCT and NET (recall Table 1).

#### Spatial variations of prediction accuracy

**Fig. 7:**
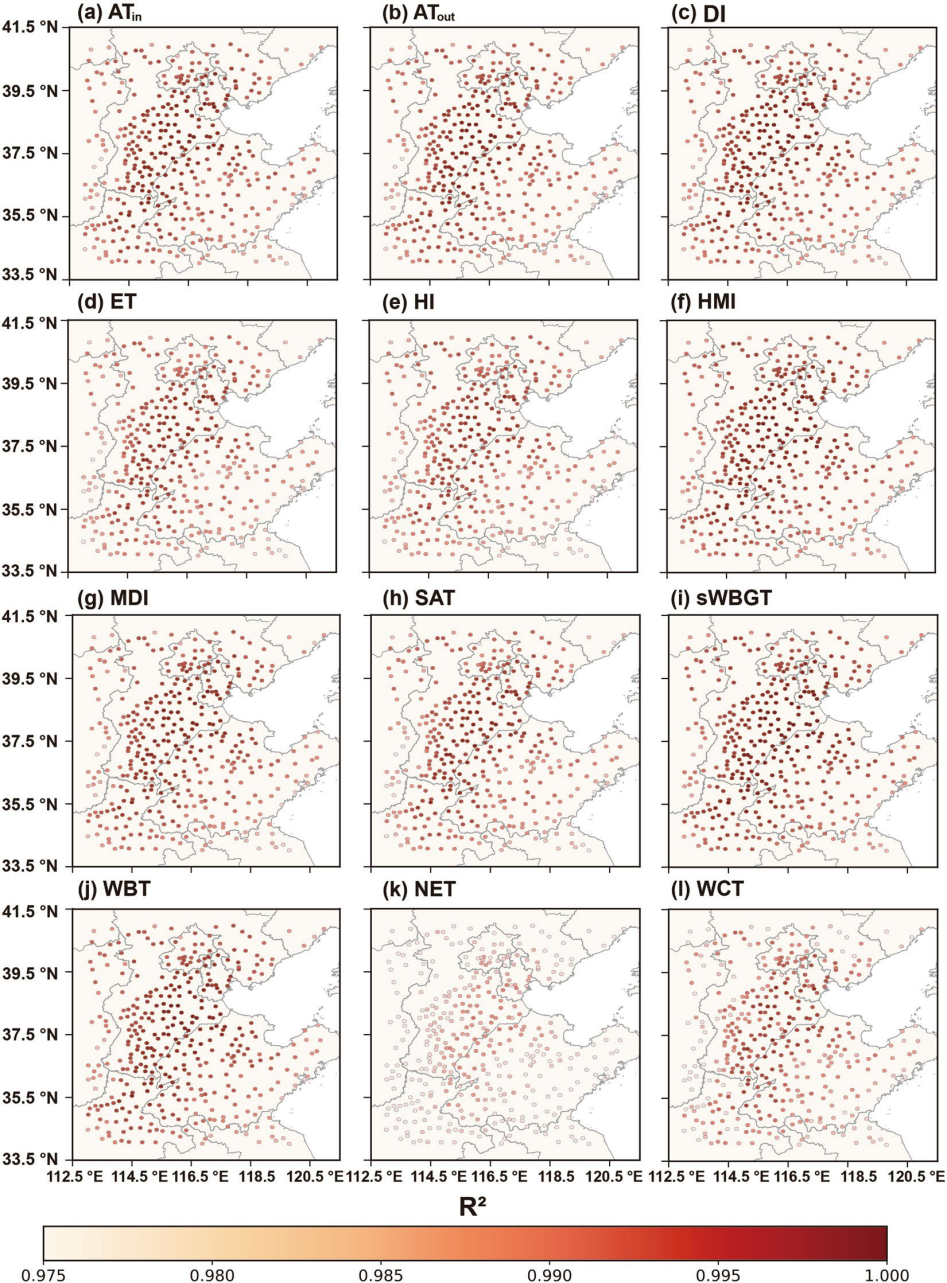
Spatial distribution of R^2^ of the 12 predicted human thermal indices at individual meteorological stations of NCP from 2003 to 2020: (**a**) AT_in_, (**b**) AT_out_, (**c**) DI, (**d**) ET, (**e**) HI, (**f**) HMI, (**g**) MDI, (**h**) SAT, (**i**) sWBGT, (**j**) WBT, (**k**) NET, and (**l**) WCT.

**Fig. 8:**
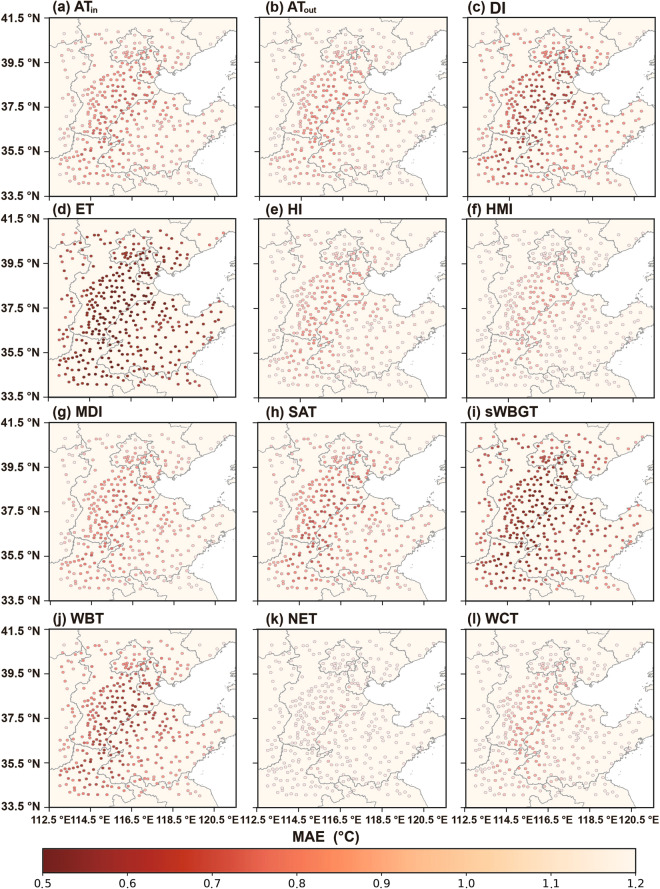
As Fig. 7 but for MAE.

**Fig. 9:**
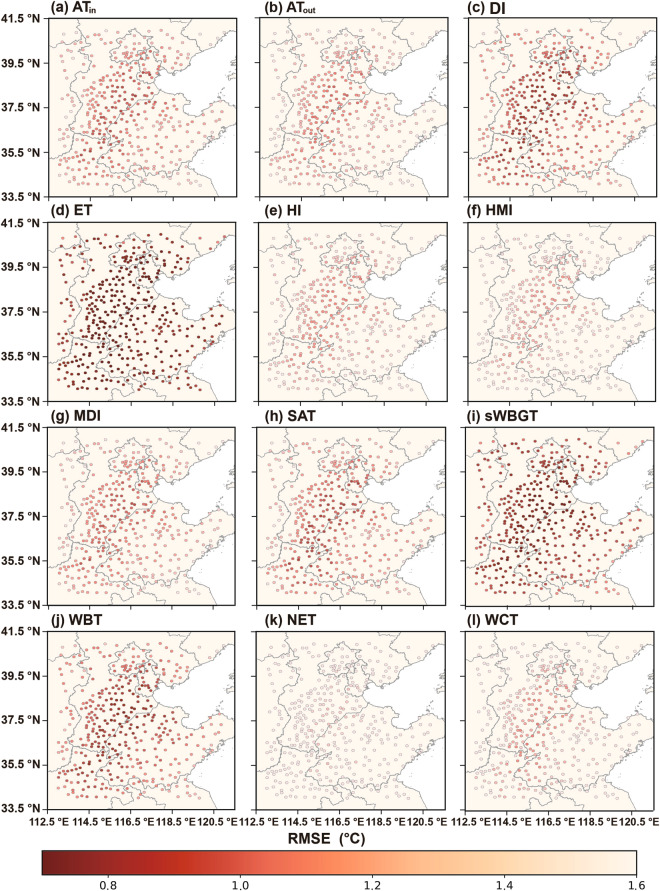
As Fig. 7 but for RMSE.

Figures 7–9 depict the distributions of R^2^, MAE, and RMSE values in 12 human thermal indices at individual stations of NCP during 2003–2020. As shown in Fig. 7, the high R^2^ values (i.e., ranging from 0.970 to 0.995) are seen at most stations across NCP, especially in the central, southwestern, and northern parts. The R^2^ values of NET at most stations have relatively lower accuracies, likely caused by the inclusion of wind speed. Figures 8, 9 show that ET has the best results with the lowest MAEs and RMSEs (i.e., < 0.911 °C and < 1.161 °C, respectively, see Figs. 8d, 9d). Compared with other 11 human thermal indices, NET has higher MAE and RMSE values (i.e., > 1.058 °C and 1.415 °C, respectively, see Figs. 8k, 9k). Spatially, MAEs and RMSEs exhibit an increasing tendency from low to high elevation, with smaller MAE and RMSE values in inland areas with lower elevation and relatively larger values in mountainous areas with higher elevation.

#### Prediction accuracy in different periods

**Fig. 10:**
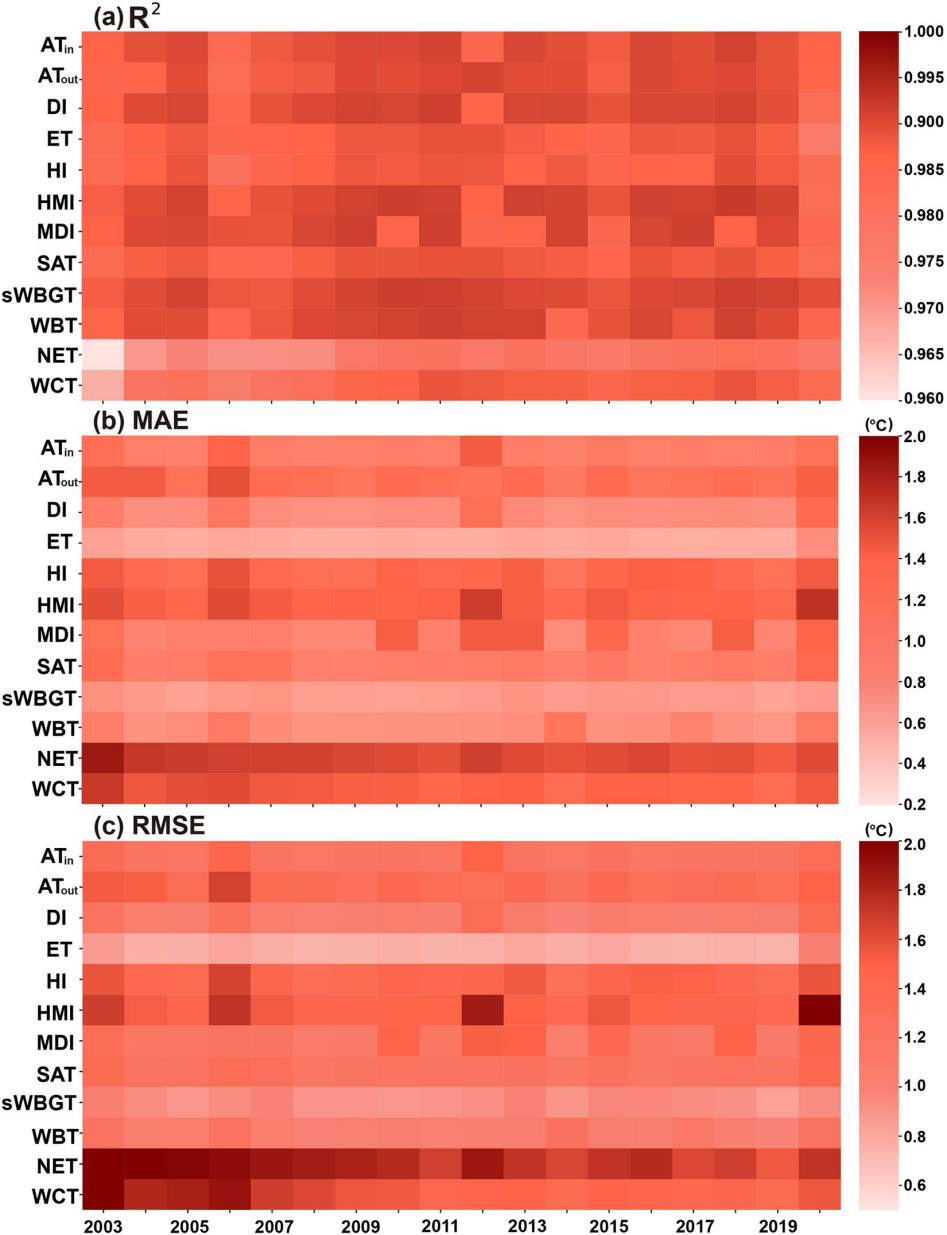
Prediction accuracies of 12 human thermal indices over NCP in individual years from 2003 to 2020.

Figure 10 illustrates the averaged R^2^, MAE, and RMSE values of 12 indices in individual years of 2003–2020. All R^2^ values of 12 indices in all years exhibit well performance (i.e., > 0.955 °C, Fig. 10a). Better annual predictions appear in WBT over 2008–2013 and sWBGT over 2008–2014, with R^2^ exceeding 0.990. The lowest R^2^ is observed in NET in 2003 (i.e., 0.956). Annual averaged MAEs and RMSEs of all indices in nearly all years are less than 1.5 °C and 2.0 °C, respectively (Fig. 10b,c), demonstrating that our product is reliable in all years.

## Data Availability

The HiTIC-NCP dataset generation codes are available on GitHub (https://github.com/CSLixiang/HiTIC-NCP.git), and operational under Python 3.8 or JavaScript. In the GitHub repository, we uploaded three code scripts, i.e., “Data preprocessing code.py”, “HiTIC-NCP Code.py” and “Figures code.py”. Additionally, the data samples were uploaded to the “Data Samples” folder.
